# Overlap of Primary Biliary Cholangitis and Systemic Sclerosis: A Case of Reynolds Syndrome

**DOI:** 10.1002/ccr3.73201

**Published:** 2026-07-24

**Authors:** Ali Gohar, Ira Abid, Syeda Eman Fatima, Abdum Muneeb Sandhu, Zain Fatima, Humayun Nasir, Fatima Arshad, Abdul Rehman Shahid Khan, Muhammad Husnain Ahmad, Masab Ali

**Affiliations:** ^1^ Lahore General Hospital Lahore Punjab Pakistan; ^2^ Department of Medicine King Edward Medical University Lahore Pakistan; ^3^ George Eliot Hospital NHS Trust Nuneaton UK; ^4^ Department of Internal Medicine Punjab Medical College Faisalabad Punjab Pakistan; ^5^ Department of Medicine Tentishev Satkynbai Memorial Asian Medical Institute Kant Kyrgyzstan

**Keywords:** autoimmune diseases, primary biliary cholangitis, Reynolds Syndrome, scleroderma

## Abstract

Reynolds Syndrome should be suspected in CREST patients with persistent cholestatic liver enzymes, since delayed diagnosis allows portal hypertension and variceal bleeding to develop. Early anti‐mitochondrial antibody screening and prompt ursodeoxycholic acid therapy improve outcomes, and these patients need multidisciplinary follow‐up for lung and thyroid disease.

## 1 Introduction

Primary biliary cholangitis (PBC) is a chronic, progressive cholestatic liver disease marked by immune‐mediated destruction of intrahepatic bile ducts, eventually leading to fibrosis and cirrhosis [1]. SSc is a connective tissue disorder with widespread microvascular injury, immune dysregulation, and excessive collagen deposition, affecting the skin and internal organs [2]. The simultaneous occurrence of these two entities is rare (Reynolds Syndrome) and poses unique diagnostic and therapeutic challenges.

Reynolds Syndrome was first described in 1971 by Telfer B. Reynolds, who recognized a subset of patients presenting with features of both PBC and scleroderma [3]. Since then, only a limited number of cases have been reported, and much of the available knowledge is derived from isolated case reports and small case series [4, 5]. The true prevalence remains uncertain, but PBC has an estimated prevalence of 20–40 cases per 100,000 population in Western countries, with significantly higher female predominance [6]. SSc has a global prevalence of approximately 7–489 cases per million, depending on geographic region [7]. Data specific to Pakistan are sparse, with only scattered reports documenting the overlap [8].

Given its rarity and complex presentation, Reynolds Syndrome remains underdiagnosed, especially in regions with limited access to advanced immunologic and hepatic testing. This case report aims to highlight the clinical spectrum, diagnostic work‐up, and management strategies for Reynolds Syndrome, emphasizing the importance of clinician awareness in improving patient outcomes.

## 2 Case Description

**FIGURE 1:**
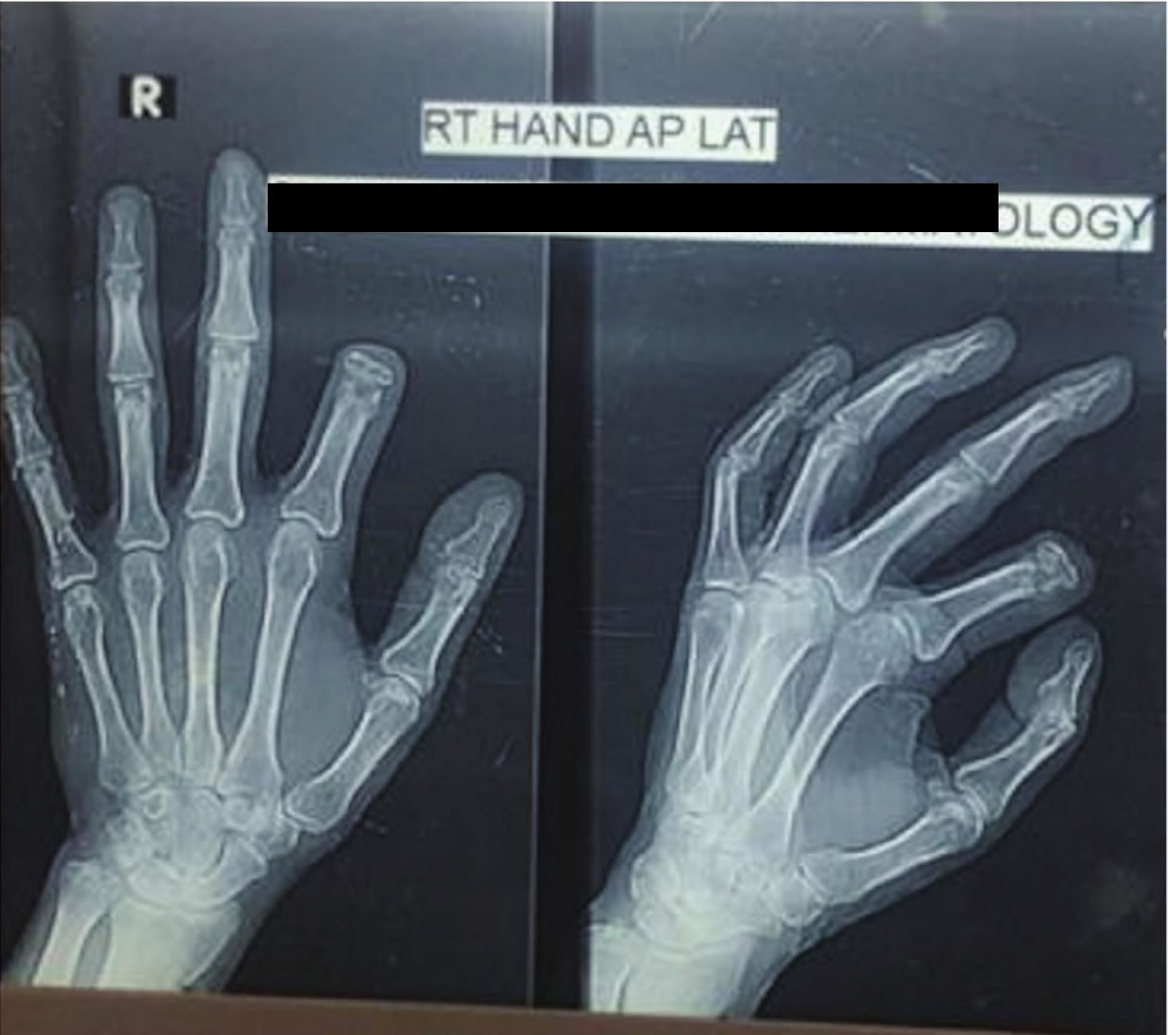
Right hand X ray showing amputated index finger due to severe digital ischemia and gangrene.

A 64‐year‐old woman presented to the rheumatology clinic with a 3‐year history of progressive skin thickening and tightening, particularly affecting her hands, face, and feet. She reported difficulty swallowing solid foods and experienced frequent episodes of discoloration of fingers and toes, often triggered by cold temperatures or stress. Physical examination revealed sclerodactyly with digital ulcers on her fingers, telangiectasias on her face and hands, and limited range of motion in her fingers and wrists. Notably, she had a history of amputation of her right‐hand index finger due to severe digital ischemia and gangrene (Figure 1). Based on history and examination, a diagnosis of calcinosis, Raynaud's phenomenon, esophageal dysmotility, sclerodactyly, and telangiectasia (CREST) syndrome was made.

**FIGURE 2:**
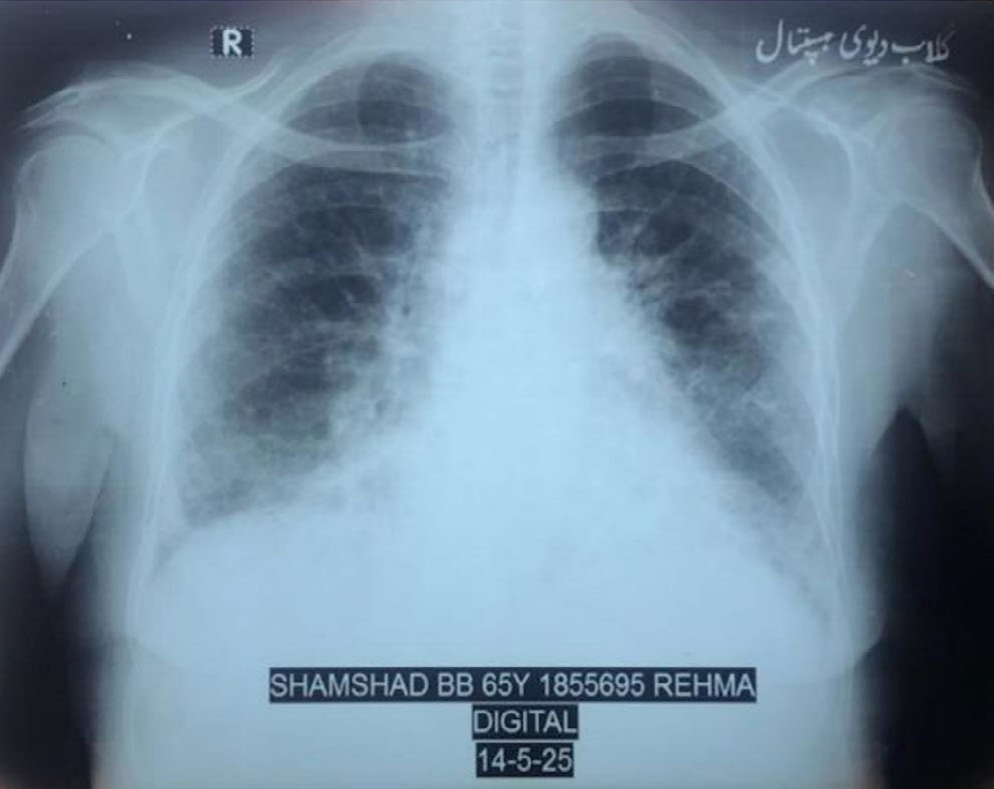
Chest X‐ray showed a fibrotic pattern in both lung fields.

Laboratory tests showed positive antinuclear antibodies (ANA) with a centromere pattern. The extractable nuclear antigen (ENA) panel showed positive anti‐centromere antibodies and negative anti‐Scl‐70, anti‐Ro, anti‐La, anti‐Smith, and anti‐ribonucleoprotein antibodies (Table 1). At baseline, her red blood cell count was 5.1 × 10^12^/L, erythrocyte sedimentation rate (ESR) was 100, C‐reactive protein (CRP) was raised, renal function tests showed creatinine 1.15 mg/dL and liver function tests (LFTs) showed alanine aminotransferase (ALT) 49 U/L, aspartate aminotransferase (AST) 90 U/L, and alkaline phosphatase (ALP) 927 U/L. The chronological evolution of laboratory findings is shown in Table 2. Urinalysis was unremarkable, and chest X‐ray showed a fibrotic pattern in both lung fields (Figure 2). Baseline viral hepatitis screening was negative, including hepatitis B surface antigen, anti‐hepatitis C virus, and human immunodeficiency virus serology. These baseline investigations are important to document systemic involvement and to exclude differential diagnoses such as autoimmune hepatitis, primary sclerosing cholangitis, nonalcoholic fatty liver disease with fibrosis, drug‐induced cholestatic liver injury, and chronic viral hepatitis. She was started on steroids and hydroxychloroquine and was asked to follow up in the rheumatology clinic. She was lost to follow‐up after 3–4 follow‐up visits.

**TABLE 1:** Autoimmune serological profile.

Test	Result	Interpretation
Anti‐Ro (Sjogren's Syndrome Antigen A)	Negative	Not suggestive of Sjögren's/Systemic Lupus Erythematosus (SLE)
Anti‐La (Sjogren's Syndrome Antigen B)	Negative	Not suggestive of Sjögren's
Anti‐Smith Antibody	Negative	Not suggestive of SLE
Anti‐Ribonucleoprotein Antibody	Negative	Not suggestive of Mixed Connective Tissue Disease (MCTD)
Anti‐Scl‐70/Anti‐Topoisomerase I Antibody	Negative	Not suggestive of diffuse Systemic Sclerosis (SSc)
Anti‐centromere	Positive	Supports CREST syndrome
Anti‐mitochondrial antibody (AMA‐M2)	Positive	Diagnostic for Primary Biliary Cholangitis (PBC)

**TABLE 2:** Longitudinal trends in laboratory investigations.

Investigation	Reference range	At first visit	2nd visit	3rd visit	4th visit
ALT	7–35 U/L	49 U/L	52 U/L	58 U/L	71 U/L
AST	10–40 U/L	90 U/L	95 U/L	104 U/L	118 U/L
ALP	44–147 U/L	927 U/L	850 U/L	760 U/L	633 U/L
GGT	9–36 U/L	28 U/L	31 U/L	38 U/L	285 U/L
Total bilirubin	0.2–1.2 mg/dL	0.8 mg/dL	0.9 mg/dL	1.0 mg/dL	1.2 mg/dL

**FIGURE 3:**
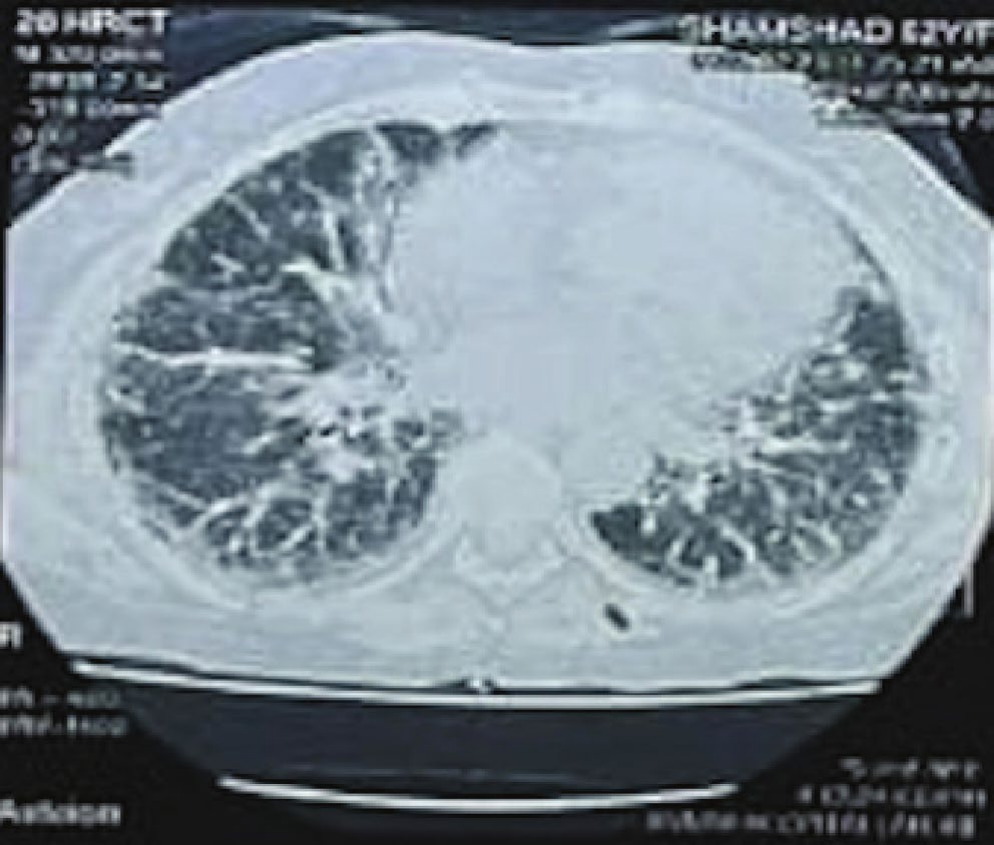
High‐resolution computed tomography (HRCT) chest suggestive of interstitial lung disease (ILD) secondary to CREST syndrome.

After approximately 1 year she again presented in the Emergency Department with complaints of shortness of breath for 1 month. On inquiry, she had discontinued her medications 2 months earlier. On examination at this time, her oxygen saturation was 94%, respiratory rate 16/min, and chest auscultation revealed decreased air entry bilaterally. Echocardiography showed calcified degenerative mitral and aortic valve and Grade 1 left ventricular (LV) dysfunction. High‐resolution computed tomography (HRCT) chest was suggestive of interstitial lung disease (ILD) (Figure 3) secondary to CREST syndrome. She was started on intravenous cyclophosphamide 750 mg/m^2^ monthly for 6 months, with monthly monitoring of blood counts and renal function, and was asked to visit pulmonology and rheumatology clinics regularly. Her condition improved during follow‐up visits.

On her follow‐up visits, her thyroid profile (thyroid‐stimulating hormone [TSH], triiodothyronine, thyroxine) was done, which showed hypothyroidism. Anti‐thyroid peroxidase (anti‐TPO) was negative. Ultrasonography (USG) neck was unremarkable. She was started on thyroxine 50 μg before breakfast daily.

After 6 months, she was presented in a medical emergency with complaints of black tarry stools for 4 days. Her ultrasound (USG) abdomen and pelvis was done, which showed a coarse texture liver with splenomegaly. Her liver function tests at this stage showed ALP 633 U/L, gamma‐glutamyl transferase (GGT) 285 U/L, bilirubin 1.2 mg/dL, and ALT/AST 71/118. Given the worsening cholestatic liver function tests, viral hepatitis workup was repeated. Serology for hepatitis A virus, hepatitis E virus, and hepatitis B virus was negative, making an infectious etiology unlikely. Upper gastrointestinal endoscopy (EGD) was done revealing esophageal varices for which endoscopic variceal band ligation (EVBL) was done, severe portal hypertensive gastropathy (PHG) and mild duodenopathy. Autoimmune liver evaluation showed positive anti‐mitochondrial (AMA‐M2) antibodies, confirming primary biliary cholangitis (PBC). She started on ursodeoxycholic acid (15 mg/kg/day) and propranolol and was advised to follow up esophagogastroduodenoscopy after 4 weeks. So, the coexistence of CREST syndrome and primary biliary cholangitis established the diagnosis of Reynolds syndrome. She was counseled for potential complications, including steroid‐induced diabetes and osteoporosis, hydroxychloroquine‐related retinal toxicity, cyclophosphamide‐induced myelosuppression and infertility, beta blocker‐related bradycardia, and gastrointestinal side effects from ursodeoxycholic acid.

## 3 Discussion

Literature review indicates a strong association between primary biliary cholangitis (PBC) and limited cutaneous systemic sclerosis (lcSSc), with a reported frequency of 2%–2.5%. Although the specific process remains unknown, genetic, environmental, and viral factors may be responsible for illness progression [9]. This cohabitation was first described in 1970 with two instances with PBC and restricted scleroderma, and was reaffirmed by Reynolds et al., who reported six patients with a similar alliance; thus, it is sometimes called PACK syndrome (primary biliary cirrhosis, anti‐centromere antibody, CREST syndrome, and keratoconjunctivitis sicca) or Reynolds Syndrome [9].

Although interstitial lung disease is more frequently associated with diffuse cutaneous systemic sclerosis, pulmonary involvement may occur in patients with limited cutaneous systemic sclerosis. In this case, the patient demonstrated the characteristic clinical features of CREST syndrome with positive anti‐centromere antibodies and negative anti‐Scl‐70 antibodies. These findings support the diagnosis of limited cutaneous systemic sclerosis, despite the presence of interstitial lung disease and highlight the heterogeneity of systemic sclerosis manifestations.

The pathophysiology of Reynolds Syndrome is thought to involve a shared autoimmune predisposition, where genetic, environmental, and epigenetic factors trigger immune‐mediated injury in both the biliary epithelium and connective tissues [4, 10]. Antimitochondrial antibodies (AMA) remain a hallmark of PBC, whereas antinuclear antibodies (ANA) and anticentromere antibodies are frequently seen in SSc. The overlap presentation may be mediated by cross‐reactive immune responses targeting antigens present in both hepatic and vascular tissues [11].

Clinically, patients may present pruritus, fatigue, and jaundice from cholestasis, alongside Raynaud's phenomenon, skin thickening, and digital ulcers from systemic sclerosis [2]. Laboratory findings include elevated ALP and GGT, with positive AMA in most cases. However, clinicians should recognize that AMA‐negative primary biliary cholangitis is a well‐described entity, and the diagnosis should be based on the overall clinical presentation, cholestatic biochemical profile, autoantibody testing, and, when appropriate, histopathological findings. Esophageal involvement is common in systemic sclerosis and, when present with portal hypertension from PBC, can predispose patients to esophageal varices, further complicating the management [1, 12].

Treatment requires a multidisciplinary approach, with ursodeoxycholic acid as the standard therapy for PBC, coupled with vasodilators, immunomodulators, and symptomatic management for SSc [2, 6]. Early recognition of the overlap is essential to prevent irreversible complications, particularly portal hypertension, pulmonary hypertension, and advanced organ fibrosis.

The clinical course of primary biliary cholangitis in patients with Reynolds syndrome may differ from that of isolated PBC, although published data is limited because of the rarity of the condition. Previous studies suggest that delayed recognition of PBC may result in progression to advanced liver disease and portal hypertensive complications before appropriate therapy is initiated. In this patient, PBC was diagnosed after the development of portal hypertension with esophageal varices and evidence of cirrhosis, emphasizing the importance of early evaluation for cholestatic liver disease in patients with systemic sclerosis who develop persistent cholestatic liver abnormalities. Earlier diagnosis may facilitate timely starting of ursodeoxycholic acid and closer follow up, which have been associated with improved biochemical response and better long‐term outcomes.

SSc is frequently associated with other autoimmune conditions, reflecting its complex immunopathogenesis. Interstitial lung disease (ILD) is one of the most common and serious complications, significantly contributing to morbidity and mortality [2]. Autoimmune thyroid disease, particularly hypothyroidism, is also well recognized among patients with SSc, suggesting a shared autoimmune background [13, 14]. In addition, PBC has a documented association with lcSSc, forming the overlap known as Reynolds Syndrome [4, 5, 9]. These associations emphasize the importance of vigilant screening and multidisciplinary management in patients with SSc to identify and address coexisting autoimmune disorders at an early stage.

## 4 Conclusion

Our case suggests a possible link between primary biliary cholangitis (PBC) and extrahepatic autoimmune disorders, such as limited cutaneous systemic sclerosis (CREST syndrome), but we also hope that it will aid in the management of patients. To avoid increased morbidity, such autoimmune illnesses should be evaluated, monitored, and followed up on a regular basis.

## Author Contributions

**Ali Gohar:** conceptualization, validation, visualization. **Ira Abid:** writing – review and editing. **Syeda Eman Fatima:** validation, visualization, writing – original draft. **Abdum Muneeb Sandhu:** conceptualization, writing – original draft. **Zain Fatima:** conceptualization, writing – original draft. **Humayun Nasir:** writing – original draft, writing – review and editing. **Fatima Arshad:** validation, visualization, writing – original draft. **Abdul Rehman Shahid Khan:** validation, visualization, writing – review and editing. **Muhammad Husnain Ahmad:** validation, writing – original draft. **Masab Ali:** validation, visualization, writing – original draft.

## Funding

The authors have nothing to report.

## Ethics Statement

The authors have nothing to report.

## Consent

Written informed consent was obtained from the patient for publication of this case report and accompanying clinical information.

## Conflicts of Interest

The authors declare no conflicts of interest.

## Data Availability

The authors have nothing to report.
